# Rate and Predictors of Patient Satisfaction After Total Joint Arthroplasty: A Cross-Sectional Study in a Low-to-Middle-Income Country

**DOI:** 10.7759/cureus.56393

**Published:** 2024-03-18

**Authors:** Moiz Ali, Fareeha Nisar, Mohammad K Safri, Manzar Abbas, Muhammad Abdullah, Haider A Lakdawala, Riaz H Lakdawala, Shahryar Noordin

**Affiliations:** 1 Orthopedic Surgery, Aga Khan University, Karachi, PAK

**Keywords:** total joint arthroplasty, low-to-middle-income country, predictors, total hip arthroplasty, total knee arthroplasty, patient satisfaction

## Abstract

Objective

This study aimed to assess the rate of patient satisfaction after primary total joint arthroplasty (TJA) using a validated satisfaction measure.

Materials and methods

A cross-sectional study was conducted, including all patients who underwent primary TJA between December 2021 and February 2023. The age of the study population was found to range from 23 to 86 years. Patient satisfaction was assessed using a validated tool comprising four questions and a quality of life (QoL) question.

Results

A total of 197 patients were included, with a mean age of 60.9 ± 12.7 years. Total knee replacement (TKR) was performed in 124 patients (62.9%), and total hip replacement (THR) in 73 patients (37.1%). The mean patient satisfaction score was 86.6 ± 14.4 out of a maximum of 100. A significant negative correlation was observed between the Charlson Comorbidity Index (CCI) and the overall satisfaction score (p-value = 0.029). The majority of the patients (52.3%, n = 103) answered that their QoL had greatly improved, and a similar level of improvement was noted in elderly vs. adult patients (p-value = 0.17). A significantly higher proportion of male patients reported improvement more than they ever expected compared to female patients, the majority of whom reported their QoL was greatly improved (p-value = 0.025).

Conclusion

Total joint arthroplasty has been shown to achieve good patient satisfaction and an improvement in QoL. However, an increased comorbidity index and female gender were identified as factors for reduced satisfaction. Hence, it is recommended to consider these factors and counsel patients accordingly based on local patient data.

## Introduction

Total knee arthroplasty (TKA) and total hip arthroplasty (THA) are successful procedures for end-stage osteoarthritis of the knee and hip, respectively [1, 2]. These are cost-effective procedures to improve functional status and quality of life (QoL). Still, they have a wide variability in reporting patient satisfaction outcomes, ranging from 70% to 95% reported in the literature [3]. This wide variability may be partly due to a lack of standardized and validated patient satisfaction measures [4]. In evaluating total joint arthroplasty (TJA), patient satisfaction is an important measure to understand its value [2,5].

In previous literature, multiple surgeons and patient-related factors have been identified that influence patient satisfaction rates after TJA [6,7]. However, a previous systematic review investigated the available literature on patient satisfaction after TJA and found that only 13% of included studies used validated patient satisfaction measures [7]. Moreover, 21.2% of the studies were unable to define how they measured satisfaction. It’s complicated to measure patient satisfaction after TJA because multiple factors influence the patient’s conclusion [7].

In the last decade, some authors have used different functional outcome scores such as the New Knee Society Score (KSS) [8], the Oxford Knee Score (OKS), the Hip Disability and Osteoarthritis Outcome Score (HOOS), the Knee Injury and Osteoarthritis Outcome Score (KOOS), and the Western Ontario and McMasters Universities Arthritis Index (WOMAC) to measure patients satisfaction [9]. On the other hand, many authors have defined satisfaction on Likert scales with variable cut-off points [10-11]. Some authors have asked multiple patient satisfaction-related questions in a questionnaire, but overall, the use of a validated satisfaction questionnaire has been rare.

Hence, the objective of this study was to assess the rate of patient satisfaction after primary TKA and THA in a low-to-middle-income country (LMIC), using a validated satisfaction measure comprising of primary questions, each rated on a five-point Likert scale, and a validated question about change in QoL [12].

## Materials and methods

A cross-sectional study was conducted at Aga Khan University Hospital, a tertiary care center in Karachi, Pakistan, where all patients who underwent primary TKA or THA between December 2021 and February 2023 were included using non-probability consecutive sampling. The age of all included patients was found to range from 23 to 86 years. Patients who required revision surgery, those with less than six months of follow-up or who had provided incomplete data, and those who did not consent to take part in the study were excluded. Approval was obtained from the institutional ethical review committee to conduct the study (approval number: 2021-6346-19712).

Basic demographic characteristics and surgical parameters for all included patients were recorded. Patients were then called over the phone six months after surgery and were asked four questions about satisfaction and one question about QoL after obtaining informed verbal consent. This set of four satisfaction questions followed by a QoL question has been validated by Goodman et al. (Cronbach’s alpha > 0.88) and published in the Journal of Arthroplasty (2020) [12].

The satisfaction tool consists of four questions, each rated using a five-point Likert scale, and a QoL question with response choice on a six-point scale (Table 1). All four questions of the satisfaction questionnaire were weighed equally. The five Likert choices were scored as 0, 25, 50, 75, and 100 in increasing order of satisfaction, and scores from each question were added and then divided by four to give the final score in the range of 0-100, with higher scores signifying better satisfaction. The answer to the QoL question (Table 2) was scored from one to six points, with high scores corresponding to poorer QoL [12].

**Table 1:** Patient satisfaction tool: How satisfied are you with the results of your knee/hip in the following areas? If you had both knees operated on, answer how you are overall.

Question: How satisfied are you with the results of your knee/hip in the following areas? If you had both knees operated on, answer how you are overall.					
	Very Satisfied	Somewhat satisfied	Neither satisfied nor dissatisfied	Somewhat dissatisfied	Very dissatisfied
For relieving pain?					
For improving your ability to do housework or yard work?					
For improving your ability to do recreational activities?					
Overall, how satisfied are you with the results of your knee surgery?					

**Table 2:** Quality of life question: How much did your knee surgery improve your quality of life?

Question: How much did your knee surgery improve your quality of life?					
More improvement than I ever dreamed possible	Great improvement	Moderate improvement	A little improvement	No improvement	The quality of my life is worse

The data were analyzed using IBM SPSS Statistics for Windows, version 23 (IBM Corp., Armonk, NY). Quantitative variables were presented as mean ± standard deviation (SD), whereas qualitative variables were expressed as percentages. An independent sample t-test, a Pearson correlation coefficient, and a Mann-Whitney U-test were used where applicable to assess for any associations between predictive factors and patient satisfaction or improvement in QoL. A p-value of <0.05 was considered significant.

## Results

This study included a total of 197 patients, with a mean age of 60.9 ± 12.7 years, and most of these patients were females (71.1%, n = 140), as shown in Table 3. The entire cohort was classified into two categories as per their age which were "elderly" (>60 years) and "adults" (<60 years), while the Charlson Comorbidity Index (CCI) was used to categorize comorbidity status, and most patients were noted to have a score of one or two (52 patients (26.4%) and 50 patients (25.4%), respectively). With regards to surgical procedures, TKR was found to be more commonly performed than total hip replacement (THR) (124 patients (62.9%) vs. 73 patients (37.1%), respectively).

**Table 3:** Baseline characteristics of the patient population

Demographics	Frequency (%)
Age group	
Adults	110 (55.8)
Elderly	87 (44.2)
Gender	
Male	57 (28.9)
Female	140 (71.1)
American Society of Anesthesiologists (ASA) class	
1	16 (8.12)
2	123 (62.4)
3	57 (28.9)
4	1 (0.51)
Charlson Comorbidity Index (CCI)	
0	29 (14.7)
1	52 (26.4)
2	50 (25.4)
3	34 (17.3)
4	22 (11.2)
5	10 (5.1)
Total hip replacement	73 (37.1)
Unilateral	68 (90.7)
Bilateral	5 (9.3)
Total knee replacement	124 (62.9)
Unilateral	37 (29.8)
Bilateral	87 (70.2)
Total knee replacement	
Patellar resurfacing	94 (75.8)
No patellar resurfacing	30 (24.2)

**Figure 1:**
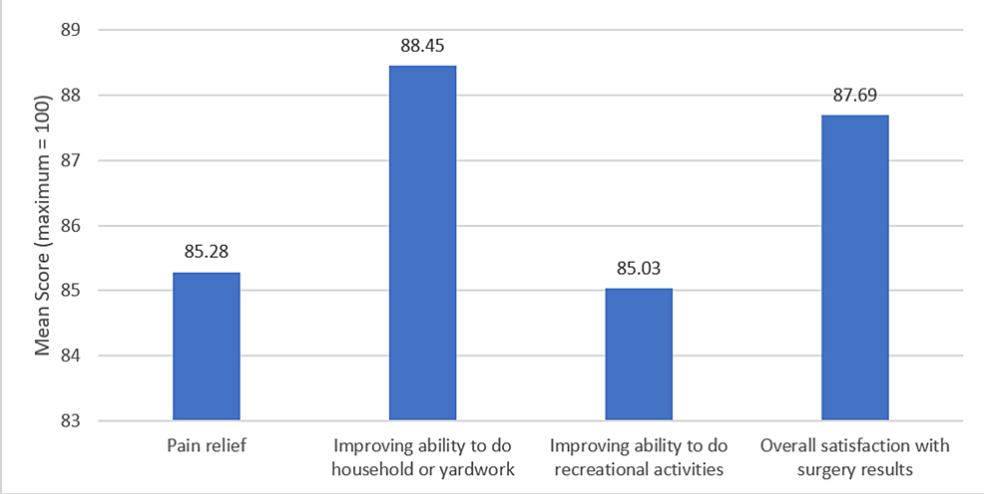
Responses to individual patient satisfaction questions

**Figure 2:**
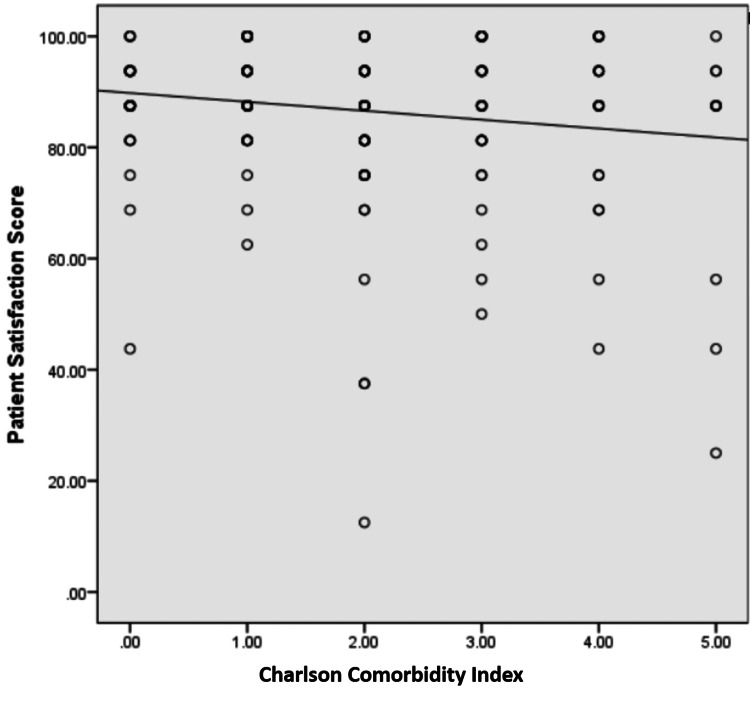
Association of the Charlson Comorbidity Index with the patient satisfaction score

**Figure 3:**
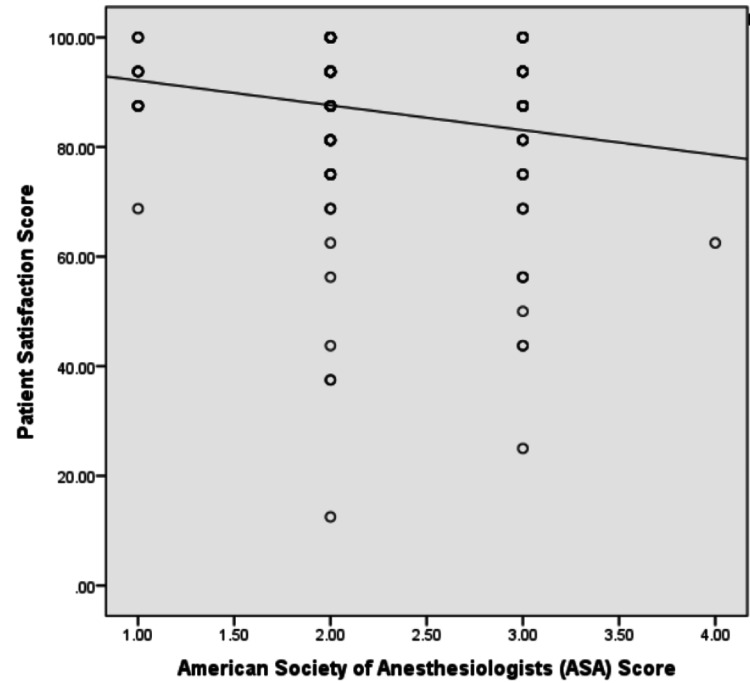
Association of the American Society of Anesthesiologists (ASA) score with the patient satisfaction score

The mean patient satisfaction score out of a maximum of 100 was noted to be 86.6 ± 14.4, with individual scores for each question mentioned in Figure 1. Mean scores were found to be similar in both age groups and genders, as shown in Table 4. A significant negative correlation was observed between CCI and the American Society of Anesthesiologists (ASA) class with the overall satisfaction score, with coefficients of -0.16 and -0.185 and p-values of 0.029 and 0.009, respectively (Figures 2-3).

**Table 4:** Association of patient demographics with the satisfaction score SD: standard deviation

Characteristics	Mean patient satisfaction (± SD)	P-value
Age group		0.19
Adults	87.8 ± 12.8	
Elderly	85.1 ± 16.1	
Gender		0.54
Male	85.6 ± 13.8	
Female	87.1 ± 14.6	

No significant difference was noted when satisfaction scores were compared after total knee or hip arthroplasty. Each procedure was further categorized into unilateral and bilateral, and subsequent comparisons in patient satisfaction also did not reveal any significant difference (Table 5). However, although not statistically significant, satisfaction after bilateral THR was noted to be higher than after unilateral THR (91.3 vs. 83.6, respectively). Patellar resurfacing was also not associated with any significant difference in patient satisfaction among patients undergoing TKR.

**Table 5:** Association of surgical procedures with the satisfaction score SD: standard deviation

Characteristics	Mean patient satisfaction (± SD)	P-value
Surgery type		0.067
Total knee replacement	88.1 ± 13.9	
Total hip replacement	84.2 ± 14.9	
Total knee replacement		0.30
Unilateral	90.0 ± 9.1	
Bilateral	87.2 ± 15.4	
Total hip replacement		0.27
Unilateral	83.6 ± 15.3	
Bilateral	91.3 ± 5.6	
Total knee replacement		0.53
Patellar resurfacing	88.5 ± 13.4	
No patellar resurfacing	86.7 ± 15.5	

Quality of life was assessed on a six-point scale, with responses ranging from "more improved than I ever imagined" to "worsened quality of life". The majority of the patients (52.3%, n = 103) answered that their QoL was "greatly improved", followed by "more improved than I ever imagined" (33.5%, n = 66) and "moderately improved" (10.2%, n = 20). While there were four patients (2.0%) who mentioned little improvement, three patients (1.5%) had no improvement, and one patient (0.5%) reported that his quality of life had worsened.

**Figure 4:**
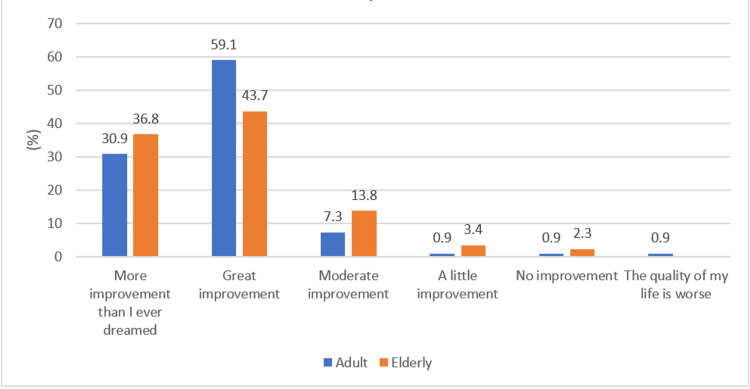
Age group-wise response to the quality of life question

**Figure 5:**
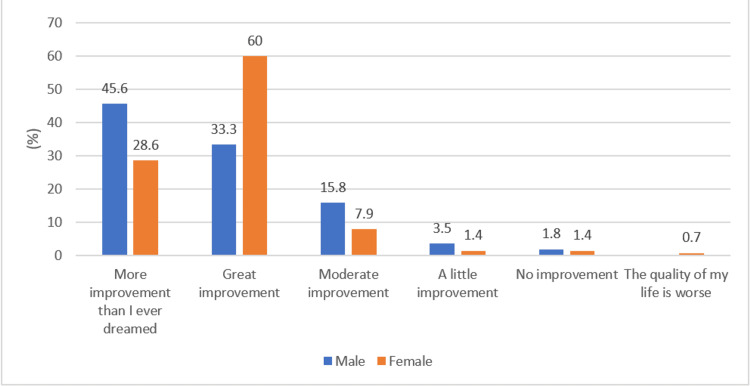
Gender-wise response to the quality of life question

Figures 4-5 show the differences in QoL when compared between the adult and elderly age groups and male and female genders, respectively. A similar level of improvement was noted in elderly vs. adult patients (p-value = 0.17). However, a significantly higher proportion of male patients reported improvement more than they ever expected compared to female patients, the majority of whom reported their QoL was greatly improved (p-value = 0.025). Furthermore, no significant association was noted between CCI and improvement in QoL (p-value = 0.85) (Table 6).

**Table 6:** Association of the Charlson Comorbidity Index (CCI) with quality of life

CCI	Quality of life (%)						P-value
	More improved than I ever dreamed	Great improvement	Moderate improvement	A little improvement	No improvement	The quality of my life is worse	
0	6 (20.7)	19 (65.5)	3 (10.3)	1 (3.4)	0	0	0.85
1	20 (38.5)	28 (53.8)	4 (7.7)	0	0	0	
2	15 (30.0)	25 (50.0)	6 (12.0)	1 (2.0)	2 (4.0)	1 (2.0)	
3	12 (35.3)	17 (50.0)	4 (11.8)	1 (2.9)	0	0	
4	9 (40.9)	10 (45.5)	2 (9.1)	0	1 (4.5)	0	
5	4 (40.0)	4 (40.0)	1 (10.0)	1 (10.0)	0	0	

**Figure 6:**
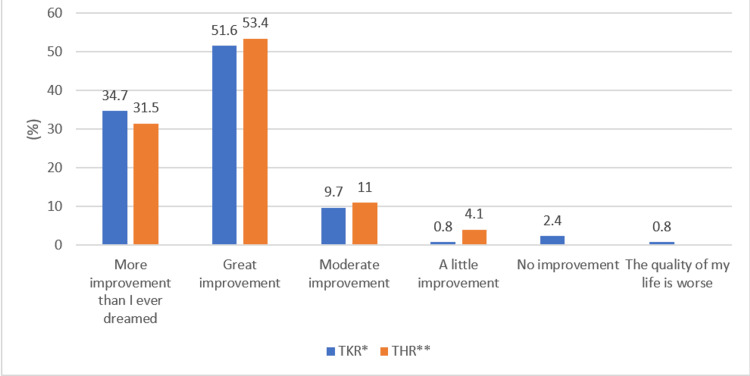
Response to the quality of life question with respect to total knee or hip replacement *TKR: total knee replacement; **THR: total hip replacement

**Figure 7:**
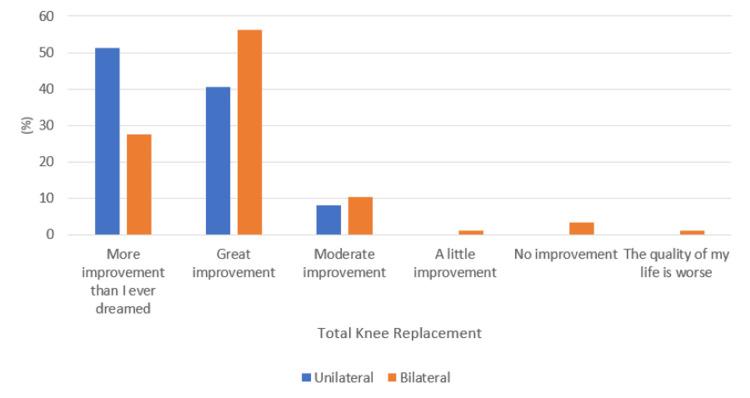
Response to the quality of life question with respect to unilateral or bilateral total knee replacement

**Figure 8:**
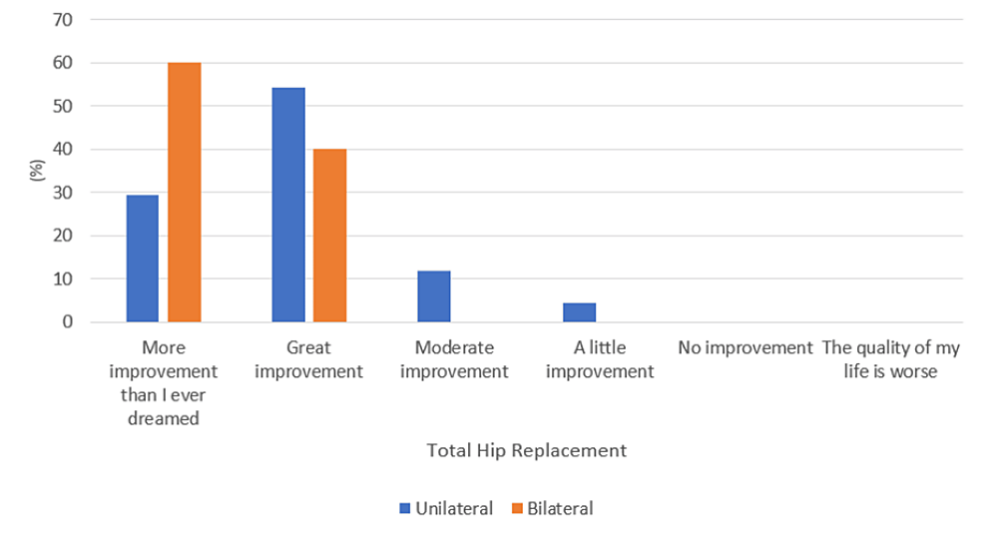
Response to the quality of life question with respect to unilateral or bilateral total hip replacement

**Figure 9:**
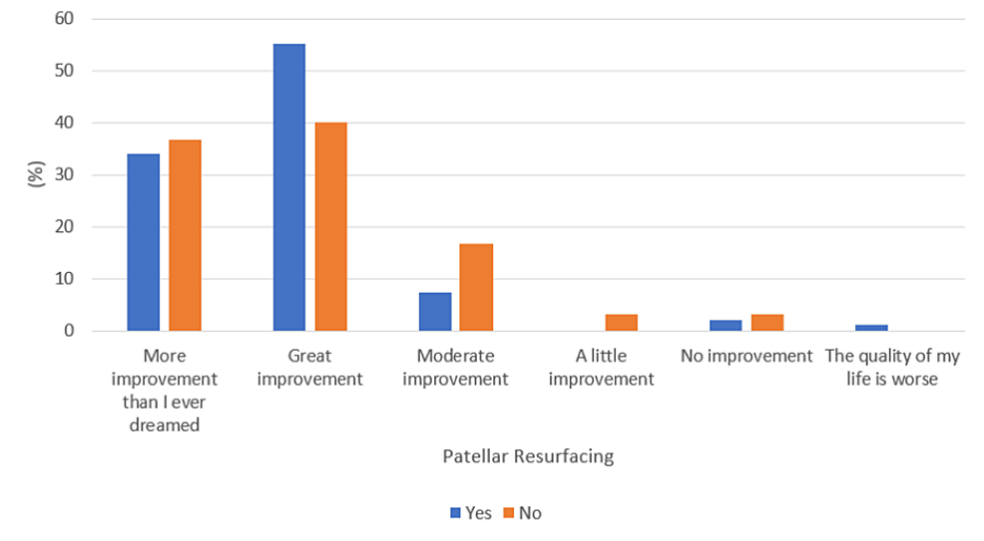
Response to the quality of life question with respect to patellar resurfacing with total knee replacement

A comparable improvement in QoL was also noted following total knee or hip replacement (p-value = 0.41), as shown in Figure 6. When analyzing whether unilateral or bilateral replacements had any impact, no significant association was noted within TKR as well as THR procedures (p-value = 0.17 and 0.50, respectively), as shown in Figures 7-8, respectively. Additionally, following TKR, no significant improvement in QoL was noted in patients who had their patella resurfaced compared to those who did not undergo resurfacing (p-value = 0.25) (Figure 9).

## Discussion

This study investigated the rate and predictors of patient satisfaction after TJA and included a comprehensive analysis of 197 patients who underwent TKR and THR in an LMIC. The main predictors analyzed were age, gender, the CCI, and unilateral or bilateral replacements.

Knee and hip replacement procedures aim to improve QoL and, as a result, achieve patient satisfaction, which becomes an important outcome measure [9]. Over the years, multiple studies have shown good patient satisfaction following both procedures, with the majority of the studies reporting satisfaction rates of >80% [9,10]. Similarly, the current study also reports an overall patient satisfaction score of 86.6 after TJA, with similar scores for TKR and THR (88.1 and 84.2, respectively).

However, there is still unconvincing evidence on the factors affecting postoperative patient satisfaction or QoL, with various studies showing different results [9]. Numerous studies have evaluated the effects of increasing age on patient satisfaction, with some studies reporting a positive correlation while others reporting a negative correlation [11, 13]. The same holds true for improvement in QoL and functional outcomes as well, with studies by Weber et al. and MacWilliam et al. showing that age is not a significant predictor of functional improvements at six-month and one-year follow-ups [1,2]. On the contrary, Nildohstter and Lormander reported that patients aged <72 years had better QoL scores at one year as compared to patients aged >72 years, which they attributed to increased comorbidities in the elderly population [3]. In accordance with this, the current study also showed a negative correlation between CCI and patient satisfaction, with a higher comorbidity index resulting in decreased satisfaction. However, no significant association was noted between satisfaction score, QoL, or age.

When evaluating patient outcomes by gender, several studies have shown that there is no significant association between total knee or hip replacement [9,14,15]. Whereas, in a systematic review by Kahlenberg et al. (2018), female gender was shown to be a factor for dissatisfaction following TKR, with male gender listed as a factor contributing towards patient satisfaction [10]. Although not associated with patient dissatisfaction, our results do show a lower level of improvement in patient-reported QoL among the female population compared to males after TJA. Cultural differences between the East and West, with women often required to do household chores that require sitting on the floor in our part of the world, maybe a contributing factor to this finding, as most surgeons advise avoiding squatting following hip or knee arthroplasty.

The distinction between unilateral and bilateral surgeries further aided in classifying patient satisfaction and improvement in QoL. It was noted that patients who underwent bilateral THR simultaneously had a higher mean satisfaction score compared to those who underwent unilateral procedures, although statistical significance was difficult to establish due to the small sample of bilateral hip replacements (n = 5). Yoshii et al. also reported significantly greater improvement in patient satisfaction following bilateral vs. unilateral THR without any increase in complication rates, thereby highlighting the effectiveness of single-staged bilateral THR in cases where required [4]. Similarly, Micicoi et al., in their study, also concluded that single-stage bilateral THR resulted in higher forgotten hip rates with similar rates of implant survival and mortality [7].

Bilateral TKR, on the other hand, was shown to have a similar patient satisfaction score as that of unilateral TKR. Previously, Putnis et al., in their cohort of 394 patients, also reported similar clinical improvement and patient satisfaction at a one-year interval following either unilateral or bilateral TKR [8]. In contrast, Sugita et al., in their study in 2020, recommended simultaneous bilateral TKR or closely staged unilateral TKR to improve patients’s QoL [5]. At our center, staged unilateral TKR is mostly limited to patients with considerable medical comorbidities who are unable to withstand prolonged anesthesia. Hence, the majority of cases of unilateral TKR were those where patients were only symptomatic on one side. We believe this is a key factor for similar QoL outcomes in both groups, as in both cohorts the symptomatic knees were replaced, providing comparable relief. Furthermore, the current study showed similar satisfaction and QoL outcomes with or without patellar resurfacing, which had previously been reported in a meta-analysis conducted by Pilling et al. [16]. However, this is still a topic without consensus, as another meta-analysis in 2021 showed improved functional scores and decreased re-operation rates following patellar resurfacing, but similar patient satisfaction, incidence of anterior knee pain, and knee range of motion [17].

Previous literature from across the world has shown similar findings to those reported in the current study; however, there is a considerable lack of data from our part of the world, where cultural and religious differences result in different lifestyle practices compared to the Western world. Therefore, the current study holds the advantage of objectively assessing and quantifying patient satisfaction and QoL in our population using a validated questionnaire, which, to the best of our knowledge, has not been previously used for our population. Furthermore, the study also highlights some of the demographic factors that may affect postoperative satisfaction levels, thus providing grounds for preoperative patient selection and counseling.

However, some of the limitations of the current study include its cross-sectional design and limited sample size, which restricted us from following patients’ outcomes over time and recording any alternate outcomes. A consecutive sampling strategy and short study duration are also identified as sources of selection bias, and administering the questionnaire over phone calls may also have led to response bias. Additionally, patient satisfaction and improvement in QoL were not correlated with functional outcome scores (e.g., Harris Hip Score or Knee Society Score), which is a consideration and recommendation for future studies. 

Furthermore, factors contributing to patient dissatisfaction following any elective procedure, including TJA, can be related to patient factors, e.g., concurrent spinal or hip disease, neurodegenerative disorder or depression, and unrealistic expectations, or technical aspects related to surgery itself, which were not evaluated separately in this study. There still exists a considerable void in the literature for analyzing patient dissatisfaction along these lines. A validated tool incorporating all these factors should allow arthroplasty surgeons to improve the rate of satisfaction and enhance the QoL after TJAs. Therefore, prospective, large-scale, multi-center studies encompassing the abovementioned factors and providing a more detailed insight into the reasons behind dissatisfaction are still warranted to reach definite conclusions and set up guidelines for preoperative patient assessment and selection in our part of the world.

## Conclusions

Total joint arthroplasty has been shown to achieve good patient satisfaction as well as improvement in quality of life internationally and in our part of the world. Although an increased comorbidity index was identified as a factor in reduced satisfaction, females were identified as reporting a lesser degree of improvement in quality of life. Hence, this study provides objective data for preoperative patient counseling in our part of the world. However, further large-scale prospective studies are required to further substantiate the findings of this study and lay out clinical guidelines in the future.
